# Supplementary material for: Interactions between Membrane Resistance, GABA-A Receptor Properties, Bicarbonate Dynamics and Cl−-Transport Shape Activity-Dependent Changes of Intracellular Cl− Concentration
Source: Int J Mol Sci. 2019 Mar 20;20(6):1416. doi: 10.3390/ijms20061416 (PMC6471822; doi:10.3390/ijms20061416)
Supplement: Supplementary file 1 [file ijms-20-01416-s001.zip › supplementary/Lombardi et al_Supplementary_Material/Lombardi et al_Suppl_Figures/Proof_Supplementary_Lombardi et al.docx]

Article

Interactions Between Membrane Resistance, GABA-A Receptor Properties, Bicarbonate Dynamics and Cl^⁻^⁻Transport Shape Activity-Dependent Changes of Intracellular Cl^⁻^ Concentration

Aniello Lombardi ^1^, Peter Jedlicka ^2,3,4^, Heiko J. Luhmann ^1^ and Werner Kilb ^1,^*

**Figure 1.** Implementation of an inward rectifying membrane conductance and estimation of the number of GABAergic inputs (n_GABA_) underlying a GDP. (**a**) Current-voltage relation recorded in a real CA3 pyramidal neurons (means ± S.E.M., black symbols) and simulation of this relation in a reconstructed neuron using inward rectifying passive conductances (grey symbols). Since membrane conductances of CA3 pyramidal neurons possess an inward rectification (black symbols, see also the original traces in the inset), we implemented a model that simulated this inward rectification (grey lines/symbols). (**b**) Relationship between GABAergic driving force (DF_Cl_) and GDP-induced [Cl^⁻^]_i_ transients (n_GABA_ = 101, g = 0.789 nS, τ = 37 ms, P_HCO3_ = 0) using the inward rectifying passive conductances (red symbols). The crosses mark values determined experimentally in real CA3 pyramidal neurons. Implementation of inward rectifying passive currents resulted, at initial [Cl^⁻^]_i_ of 10 mM, in GDP-induced [Cl^⁻^]_i_ changes of 0.32 mM when using g_pas_ of 0.049 nS/cm^2^ and of 0.27 mM with the model using voltage-dependent g_Input_. In contrast, at an initial [Cl^⁻^]_i_ of 50 mM, which results in strongly depolarizing GABAergic responses, an obvious discrepancy between passive (1.64 mM) and voltage⁻dependent g_Input_ model (3.8 mM) was observed. These transients are still smaller than the experimentally determined GDP-induced [Cl^⁻^]_i_ transients observed in real CA3 pyramidal neurons. (**c**) Holding current (I_Hold_) and input resistance (R_Input_) during a simulated GDP (n_GABA_ = 101, g = 0.789 nS, τ = 37 ms, P_HCO3_ = 0, [Cl^⁻^]_i_ = 10 mM) under voltage⁻clamp conditions at a holding potential (V_Hold_) of −60 mV. Note the massive decrease in R_Input_ during a GDP, which will impede the estimation of n_GABA_. Accordingly, the charge transfer during a simulated GDP in the reconstructed neuron using similar experimental paradigms as in the real neurons ([Cl^⁻^]_i_ = 10 mM, V_Hold_ = 0 mV) revealed that 101 GABAergic synapses induced an average charge transfer of 43.1 pC, which was substantially less than the charge transfer of 88.3 ± 3 pC determined in real CA3 pyramidal neurons under this condition. (**d**) GDP⁻induced membrane currents recorded at V_Hold_ of 0 mV in a real CA3 pyramidal neuron (gray trace) and simulated GDP⁻induced membrane currents in the reconstructed neuron with n_GABA_ of 101 (green), 201 (blue) and 301 (red). (**e**) Relationship between DF_Cl_ and GDP-induced [Cl^⁻^]_i_ transients obtained with different n_GABA_ of 101 (green), 201 (blue) and 301 (red) using a P_HCO3_ of 0. While increasing n_GABA_ to 301 resembles the experimentally observed change transfer, it is evident from these simulations that the obtained maximal [Cl^⁻^]_i_ changes of 2.7 mM, 3.8 mM, and 4.3 mM (for n_GABA_ =101, 201, and 301, respectively) are still lower than the maximal [Cl^⁻^]_i_ changes of 15.7 ± 4.7 mM (*n* = 6) determined experimentally at comparable [Cl^⁻^]_i_ levels (ca. 50 mM) in real CA3 pyramidal neurons. (**f**) GDP-induced charge transfer at V_Hold_ of 0 mV using different n_GABA_. The different shades of purple reflect P_HCO3_ values of 0.0, 0.18 and 0.44, respectively. Since the space clamp problem is influenced by the membrane depolarization, which in turn critically depends on P_HCO3_ (see 2.3), we also determined the number of GABAergic synapses (n_GABA_) that need to be implemented to simulate the observed charge transfer at a P_HCO3_ of 0.18 [48] and 0.44 [49]. These experiments revealed that implementation of 302, 395 (black dashed lines) and 523 GABAergic synapses (for P_HCO3_ values of 0.0, 0.18, and 0.44, respectively) was required to simulate the charge transfer of 88.3 pC.

**Figure 2.** Influence of the GABAergic conductance (g_GABA_) and the decay kinetics of GABA_A_ receptor mediated currents (τ_GABA_) on GABA-induced [Cl^⁻^]_i_ transients. (**a**) Spatial profile of [Cl^⁻^]_i_ upon a single synaptic stimulation (g_GABA_ = 3.945 nS, τ = 37 ms, P_HCO3_ = 0, [Cl^⁻^]_i_ = 30 mM, at 100 µm) obtained at different times after stimulation in an isolated dendrite, the different shades (from dark to light) represent values obtained at a distance of 0 µm, 50 µm and 100 µm from the synapse, respectively. (**b**) Time course of [Cl^⁻^]_i_ upon a single synaptic stimulation using g_GABA_ of 3.945 nS (blue lines) and 39.45 nS (red lines). Note that [Cl^⁻^]_i_ was evenly distributed within the dendrite after 3 s. Therefore, we estimated the total amount of GABA-evoked [Cl^⁻^]_i_ changes by averaging the [Cl^⁻^]_i_ over all nodes of the dendrite 3 s after the GABAergic stimulus. (**c**) Time course of E_m_ upon a single synaptic stimulation (g_GABA_ = 0.789 nS, P_HCO3_ = 0, [Cl^⁻^]_i_ = 30 mM) in an isolated dendrite, the different shades (from dark to light) represent simulations using τ_GABA_ values of 10, 100 and 1000 ms, respectively. (**d**) Time course of [Cl^⁻^]_i_ upon a single synaptic stimulation at different τ_GABA_. Note the strong effect of τ_GABA_ on [Cl^⁻^]_i._ As expected, increasing τ_GABA_ prolonged the depolarization and enhanced the [Cl^⁻^]_i_ transients.

**Figure 3.** Influence of the relative HCO_3_^⁻^ conductivity (P_HCO3_) on GABA-induced membrane depolarization and [Cl^⁻^]_i_ transients at constant and variable [HCO_3_^⁻^]_i_. (**a**) Dependency between peak depolarization and [Cl^⁻^]_i_ upon a single synaptic stimulation (g_GABA_ = 7.89 nS, τ = 37 ms) at P_HCO3_ of 0.0 (red), 0.18 (blue) and 0.44 (green) under the assumption of a constant [Cl^⁻^]_i_ of 30 mM. Implementing HCO_3_^⁻^ permeability in GABA_A_ receptors shifted, as expected, the [Cl^⁻^]_i_ voltage relation from a purely linear behavior (according to the Nernst equation) to the function described by the Goldman-Hodgkin-Katz equation. (**b**) Dependency between peak depolarization and [Cl^⁻^]_i_ upon a single synaptic stimulation (g_GABA_ = 7.89 nS, τ = 37 ms, initial [Cl^⁻^]_i_ = 30 mM) at different P_HCO3_ after implementation of dynamic [Cl^⁻^]_i_. (with constant [HCO_3_^⁻^]_i_ of 14.1 mM). When the static [Cl^⁻^]_i_ model was replaced with a dynamic [Cl^⁻^]_i_ model, the [Cl^⁻^]_i_ fluxes during activation of GABA_A_ receptors affect E_GABA_. In consequence, the relation between [Cl^⁻^]_i_ and E_m_ was non-linear even at P_HCO3_ of 0. For P_HCO3_ of 0.18 and 0.44 even larger deviations in E_m_ between static and dynamic [Cl^⁻^]_i_ conditions were observed. This attenuation of depolarization was caused by the fact, that the GABAergic HCO_3_^⁻^ currents mediate an additional depolarizing component. (**c**) Time course of E_m_ (in mV) and [HCO_3_^⁻^]_i_ (in mM) changes upon a single synaptic stimulation (g_GABA_ = 7.89 nS, τ = 37 ms) in an isolated dendrite with static/dynamic [HCO_3_^⁻^]_i_ at different P_HCO3_. Shaded lines represent results obtained with a constant [HCO_3_^⁻^]_i_ of 14.1 mM, while plain lines represent results obtained with a model that simulates HCO_3_^⁻^ fluxes. Note that the implementation of dynamic [HCO_3_^⁻^]_i_  (plain lines) massively reduces the peak depolarization as compared to conditions with static [HCO_3_^⁻^]_i_  (shaded lines). (**d**) Effect of increasing g_GABA_ on GABA-induced [Cl^⁻^]_i_ changes at different P_HCO3_ for an initial [Cl^⁻^]_i_ of 30 mM in an isolated dendrite. Note that the implementation of dynamic [HCO_3_^⁻^]_i_  (plain lines) reduces the maximal [Cl^⁻^]_i_ changes, as compared to conditions with static [HCO_3_^⁻^]_i_  (shaded lines). Upon intense synaptic stimulation (g_GABA_ = 789 nS) the maximal [Cl^⁻^]_i_ change turns from an influx of 6.7 mM at stable [HCO_3_^⁻^]_i_ to an efflux of 0.9 mM under dynamic [HCO_3_^⁻^]_i_ at a P_HCO3_ of 0.18. For a P_HCO3_ of 0.44, stable [HCO_3_^⁻^]_i_ conditions led to an influx of 8.5 mM, while dynamic [HCO_3_^⁻^]_i_ let to an efflux of 0.7 mM. Thus dynamic [HCO_3_^⁻^]_i_  conditions can even reverse the direction of Cl^⁻^ fluxes. In addition, these simulations revealed that with dynamic [HCO_3_^⁻^]_i_  the GABA-induced [Cl^⁻^]_i_ changes soon reach saturation. (**e**) [HCO_3_^⁻^]_i_ changes induced in a reconstructed CA3 pyramidal neuron by a simulated GDP (g_GABA_ = 0.789 nS, τ = 37 ms, initial [Cl^⁻^]_i_ = 30 mM, initial [HCO_3_^⁻^]_i_ = 14.1) at different initial [Cl^⁻^]_i_ and P_HCO3_ under dynamic [HCO_3_^⁻^]_i_  conditions. Note that GDPs induce considerable [HCO_3_^⁻^]_i_ changes, and that the amount of [HCO_3_^⁻^]_i_ changes was smaller at high [Cl^⁻^]_i_.

**Figure 4.** Influence of the stability of HCO_3_^⁻^ gradients, pH and Cl^⁻^-transport kinetics on GABA-induced membrane depolarization and [Cl^⁻^]_i_ transients. (**a**) Time course of [Cl^⁻^]_i_ (in mM) and [HCO_3_^⁻^]_i_ (in mM) upon a single synaptic stimulation (g_GABA_ = 7.89 nS, τ = 37 ms, P_HCO3_ = 0.18, initial [Cl^⁻^]_i_ = 30 mM) in an isolated dendrite at different τ_HCO3_ (indicated by the shadings). Note that while [HCO_3_^⁻^]_i_ was massively affected by τ_HCO3_, [Cl^⁻^]_i_ was only altered at very fast τ_HCO3_. (**b**) Dependency between maximal [HCO_3_^⁻^]_i_ changes and τ_HCO3_ at P_HCO3_ of 0.18 and 0.44 upon a single synaptic stimulation (g_GABA_ = 7.89 nS, τ = 37 ms, initial [Cl^⁻^]_i_ = 30 mM). Note the sigmoidal dependency between both parameters with halfmaximal responses obtained at τ_HCO3_ of ca. 70 ms. (**c**) Time course of GDP-induced [Cl^⁻^]_i_ and [HCO_3_^⁻^]_i_ changes (in mM) in the reconstructed CA3 pyramidal neuron (g_GABA_ = 0.789 nS, τ = 37 ms, P_HCO3_ = 0.18, n_GABA_ = 395, initial [Cl^⁻^]_i_ = 30 mM) at different τ_HCO3_. (**d**) Dependency between GDP-induced [HCO_3_^⁻^]_i_ changes and initial [Cl^⁻^]_i_ for different τ_HCO3_. The GDP-induced [HCO_3_^⁻^]_i_ changes were virtually absent at τ_HCO3_ of 1 ms (0.15 mM) and amounted to 3.4 mM, 4.5 mM and 4.8 mM for τ_HCO3_ values of 90 ms, 518 ms and 3 s, respectively. (**e**) Dependency between peak GDP-induced depolarizations (g_GABA_ = 0.789 nS, τ = 37 ms, P_HCO3_ = 0.18, n_GABA_ = 395, τ_HCO3 = 1s_) and initial [Cl^⁻^]_i_ for different pH in the reconstructed CA3 pyramidal neuron. Note that the peak E_m_ was shifted to depolarized potentials with alkaline pH. (**f**–**h**) Time course of [Cl^⁻^]_i_ upon a single synaptic stimulation (g_GABA_ = 1.28 µS, τ = 37 ms, P_HCO3_ = 0.18, initial [Cl^⁻^]_i_ = 10 mM) determined at the subsynaptic site (red lines) and at the proximal end of an dendrite (blue lines). [Cl^⁻^]_i_ transients were simulated either in a isolated dendrite (l = 200 µm, shaded lines) or in the identical dendrite connected to a soma (Ø 20 µm, bright lines) at τ_Cl_ of 321 s (**f**), 10 s (**g**) and 1 s (**h**). Note that for τ_Cl_ of 321 s and 10 s the [Cl^⁻^]_i_ recovers only in the soma-attached configuration, while at τ_Cl_ = 1 s the [Cl^⁻^]_i_ recovery has a similar decay, suggesting dominant Cl^⁻^ elimination via the dendritic membrane. (**i**–**k**) Distribution of [Cl^⁻^]_i_ along a 200 µm long dendrite attached to a soma (Ø 20 µm) determined 25 ms, 200 ms, 1 s, and 4 s after the synaptic stimulus (parameters as in a) for τ_Cl_ of 321 s (**i**), 10 s (**j**) and 1 s (**k**). Note that the [Cl^⁻^]_i_ in distant parts of the dendrite was substantially lower for t ≥ 1s at τ_Cl_ of 10 s and 1 s, as compared to τ_Cl_ of 321 s. Please also note that for τ_Cl_ ≥ 10s [Cl^⁻^]_i_ was substantial lower in the proximal than in the distal part of the dendrite, suggesting a dominance of diffusional elimination of Cl^⁻^ towards the soma under these conditions.
