# Supplementary material for: Interactions between Membrane Resistance, GABA-A Receptor Properties, Bicarbonate Dynamics and Cl−-Transport Shape Activity-Dependent Changes of Intracellular Cl− Concentration
Source: Int J Mol Sci. 2019 Mar 20;20(6):1416. doi: 10.3390/ijms20061416 (PMC6471822; doi:10.3390/ijms20061416)
Supplement: Supplementary file 1 [file ijms-20-01416-s001.zip › supplementary/Lombardi et al_Supplementary_Material/Lombardi et al_Suppl_Figures/Proof_Supplementary_Lombardi et al.pdf]

International Journal of *Molecular Sciences*

MDPI

Comment [ww1]: Ok ???

Article

Interactions Between Membrane Resistance, GABA-A Receptor Properties, Bicarbonate Dynamics and Cl⁻-Transport Shape Activity-Dependent Changes of Intracellular Cl⁻ Concentration

Aniello Lombardi [1], Peter Jedlicka [2,3,4], Heiko J. Luhmann [1] and Werner Kilb [1,*]

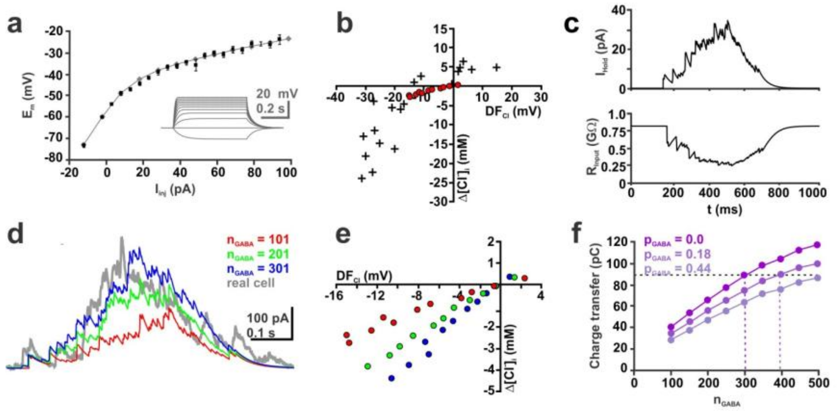

Figure 1. Implementation of an inward rectifying membrane conductance and estimation of the number of GABAergic inputs (n_{GABA}) underlying a GDP. (**a**) Current-voltage relation recorded in a real CA3 pyramidal neurons (means ± S.E.M., black symbols) and simulation of this relation in a reconstructed neuron using inward rectifying passive conductances (grey symbols). Since membrane conductances of CA3 pyramidal neurons possess an inward rectification (black symbols, see also the original traces in the inset), we implemented a model that simulated this inward rectification (grey lines/symbols). (**b**) Relationship between GABAergic driving force (DF_{Cl}) and GDP-induced $[Cl^-]_i$ transients (n_{GABA} = 101, g = 0.789 nS, τ = 37 ms, P_{HCO3} = 0) using the inward rectifying passive conductances (red symbols). The crosses mark values determined experimentally in real CA3 pyramidal neurons. Implementation of inward rectifying passive currents resulted, at initial $[Cl^-]_i$ of 10 mM, in GDP-induced $[Cl^-]_i$ changes of 0.32 mM when using g_{pas} of 0.049 nS/cm² and of 0.27 mM with the model using voltage-dependent g_{Input}. In contrast, at an initial $[Cl^-]_i$ of 50 mM, which results in strongly depolarizing GABAergic responses, an obvious discrepancy between passive (1.64 mM) and voltage-dependent g_{Input} model (3.8 mM) was observed. These transients are still smaller than the experimentally determined GDP-induced $[Cl^-]_i$ transients observed in real CA3 pyramidal neurons. (**c**) Holding current (I_{Hold}) and input resistance (R_{Input}) during a simulated GDP (n_{GABA} = 101, g = 0.789 nS, τ = 37 ms, P_{HCO3} = 0, $[Cl^-]_i$ = 10 mM) under voltage-clamp conditions at a holding potential (V_{Hold}) of −60 mV. Note the massive decrease in R_{Input} during a GDP, which will impede the estimation of n_{GABA}. Accordingly, the charge transfer during a simulated GDP in the reconstructed neuron using similar experimental paradigms as in the real neurons ($[Cl^-]_i$ = 10 mM, V_{Hold} = 0 mV) revealed that 101 GABAergic synapses induced an average charge transfer of 43.1 pC, which was substantially less than the charge transfer of 88.3 ± 3 pC determined in real CA3 pyramidal neurons under this condition. (**d**) GDP-induced membrane currents recorded at V_{Hold} of 0 mV in a real CA3 pyramidal neuron (gray trace) and simulated GDP-induced membrane currents in the reconstructed neuron

with n_{GABA} of 101 (green), 201 (blue) and 301 (red). (**e**) Relationship between DF_{Cl} and GDP-induced $[Cl^-]_i$ transients obtained with different n_{GABA} of 101 (green), 201 (blue) and 301 (red) using a P_{HCO3} of 0. While increasing n_{GABA} to 301 resembles the experimentally observed change transfer, it is evident from these simulations that the obtained maximal $[Cl^-]_i$ changes of 2.7 mM, 3.8 mM, and 4.3 mM (for n_{GABA} =101, 201, and 301, respectively) are still lower than the maximal $[Cl^-]_i$ changes of 15.7 ± 4.7 mM (n = 6) determined experimentally at comparable $[Cl^-]_i$ levels (ca. 50 mM) in real CA3 pyramidal neurons. (**f**) GDP-induced charge transfer at V_{Hold} of 0 mV using different n_{GABA}. The different shades of purple reflect P_{HCO3} values of 0.0, 0.18 and 0.44, respectively. Since the space clamp problem is influenced by the membrane depolarization, which in turn critically depends on P_{HCO3} (see 2.3), we also determined the number of GABAergic synapses (n_{GABA}) that need to be implemented to simulate the observed charge transfer at a P_{HCO3} of 0.18 [48] and 0.44 [49]. These experiments revealed that implementation of 302, 395 (black dashed lines) and 523 GABAergic synapses (for P_{HCO3} values of 0.0, 0.18, and 0.44, respectively) was required to simulate the charge transfer of 88.3 pC.

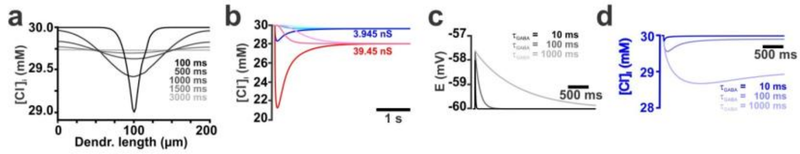

Figure 2. Influence of the GABAergic conductance (g_{GABA}) and the decay kinetics of $GABA_A$ receptor mediated currents (τ_{GABA}) on GABA-induced $[Cl^-]_i$ transients. (**a**) Spatial profile of $[Cl^-]_i$ upon a single synaptic stimulation (g_{GABA} = 3.945 nS, τ = 37 ms, P_{HCO3} = 0, $[Cl^-]_i$ = 30 mM, at 100 µm) obtained at different times after stimulation in an isolated dendrite, the different shades (from dark to light) represent values obtained at a distance of 0 µm, 50 µm and 100 µm from the synapse, respectively. (**b**) Time course of $[Cl^-]_i$ upon a single synaptic stimulation using g_{GABA} of 3.945 nS (blue lines) and 39.45 nS (red lines). Note that $[Cl^-]_i$ was evenly distributed within the dendrite after 3 s. Therefore, we estimated the total amount of GABA-evoked $[Cl^-]_i$ changes by averaging the $[Cl^-]_i$ over all nodes of the dendrite 3 s after the GABAergic stimulus. (**c**) Time course of E_m upon a single synaptic stimulation (g_{GABA} = 0.789 nS, P_{HCO3} = 0, $[Cl^-]_i$ = 30 mM) in an isolated dendrite, the different shades (from dark to light) represent simulations using τ_{GABA} values of 10, 100 and 1000 ms, respectively. (**d**) Time course of $[Cl^-]_i$ upon a single synaptic stimulation at different τ_{GABA}. Note the strong effect of τ_{GABA} on $[Cl^-]_i$. As expected, increasing τ_{GABA} prolonged the depolarization and enhanced the $[Cl^-]_i$ transients.

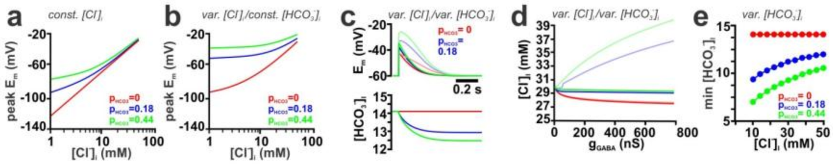

Figure 3. Influence of the relative HCO_3^- conductivity (P_{HCO3}) on GABA-induced membrane depolarization and $[Cl^-]_i$ transients at constant and variable $[HCO_3^-]_i$. (**a**) Dependency between peak depolarization and $[Cl^-]_i$ upon a single synaptic stimulation (g_{GABA} = 7.89 nS, τ = 37 ms) at P_{HCO3} of 0.0 (red), 0.18 (blue) and 0.44 (green) under the assumption of a constant $[Cl^-]_i$ of 30 mM. Implementing HCO_3^- permeability in $GABA_A$ receptors shifted, as expected, the $[Cl^-]_i$ voltage relation from a purely linear behavior (according to the Nernst equation) to the function described by the Goldman-Hodgkin-Katz equation. (**b**) Dependency between peak depolarization and $[Cl^-]_i$ upon a single synaptic stimulation (g_{GABA} = 7.89 nS, τ = 37 ms, initial $[Cl^-]_i$ = 30 mM) at different P_{HCO3} after implementation of dynamic $[Cl^-]_i$. (with constant $[HCO_3^-]_i$ of 14.1 mM). When the static $[Cl^-]_i$ model was replaced with a dynamic $[Cl^-]_i$ model, the $[Cl^-]_i$ fluxes during activation of $GABA_A$ receptors affect E_{GABA}. In consequence, the relation between $[Cl^-]_i$ and E_m was non-linear even at P_{HCO3} of 0. For P_{HCO3} of 0.18 and 0.44 even larger deviations in E_m between static and dynamic $[Cl^-]_i$ conditions were observed. This attenuation of depolarization was caused by the fact, that the GABAergic HCO_3^-

currents mediate an additional depolarizing component. (**c**) Time course of E_m (in mV) and $[HCO_3^-]_i$ (in mM) changes upon a single synaptic stimulation (g_{GABA} = 7.89 nS, τ = 37 ms) in an isolated dendrite with static/dynamic $[HCO_3^-]_i$ at different P_{HCO3}. Shaded lines represent results obtained with a constant $[HCO_3^-]_i$ of 14.1 mM, while plain lines represent results obtained with a model that simulates HCO_3^- fluxes. Note that the implementation of dynamic $[HCO_3^-]_i$ (plain lines) massively reduces the peak depolarization as compared to conditions with static $[HCO_3^-]_i$ (shaded lines). (**d**) Effect of increasing g_{GABA} on GABA-induced $[Cl^-]_i$ changes at different P_{HCO3} for an initial $[Cl^-]_i$ of 30 mM in an isolated dendrite. Note that the implementation of dynamic $[HCO_3^-]_i$ (plain lines) reduces the maximal $[Cl^-]_i$ changes, as compared to conditions with static $[HCO_3^-]_i$ (shaded lines). Upon intense synaptic stimulation (g_{GABA} = 789 nS) the maximal $[Cl^-]_i$ change turns from an influx of 6.7 mM at stable $[HCO_3^-]_i$ to an efflux of 0.9 mM under dynamic $[HCO_3^-]_i$ at a P_{HCO3} of 0.18. For a P_{HCO3} of 0.44, stable $[HCO_3^-]_i$ conditions led to an influx of 8.5 mM, while dynamic $[HCO_3^-]_i$ let to an efflux of 0.7 mM. Thus dynamic $[HCO_3^-]_i$ conditions can even reverse the direction of Cl^- fluxes. In addition, these simulations revealed that with dynamic $[HCO_3^-]_i$ the GABA-induced $[Cl^-]_i$ changes soon reach saturation. (**e**) $[HCO_3^-]_i$ changes induced in a reconstructed CA3 pyramidal neuron by a simulated GDP (g_{GABA} = 0.789 nS, τ = 37 ms, initial $[Cl^-]_i$ = 30 mM, initial $[HCO_3^-]_i$ = 14.1) at different initial $[Cl^-]_i$ and P_{HCO3} under dynamic $[HCO_3^-]_i$ conditions. Note that GDPs induce considerable $[HCO_3^-]_i$ changes, and that the amount of $[HCO_3^-]_i$ changes was smaller at high $[Cl^-]_i$.

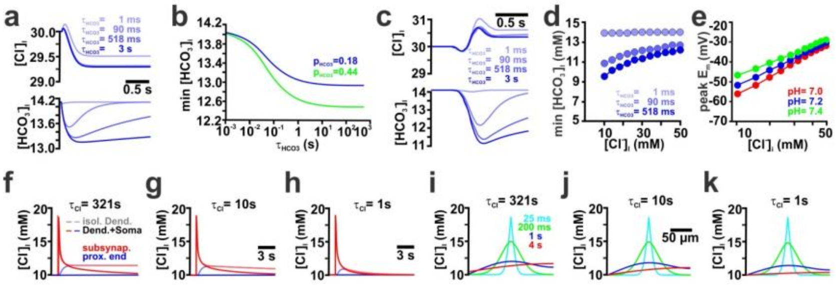

Figure 4. Influence of the stability of HCO_3^- gradients, pH and Cl^--transport kinetics on GABA-induced membrane depolarization and $[Cl^-]_i$ transients. (**a**) Time course of $[Cl^-]_i$ (in mM) and $[HCO_3^-]_i$ (in mM) upon a single synaptic stimulation (g_{GABA} = 7.89 nS, τ = 37 ms, P_{HCO3} = 0.18, initial $[Cl^-]_i$ = 30 mM) in an isolated dendrite at different $τ_{HCO3}$ (indicated by the shadings). Note that while $[HCO_3^-]_i$ was massively affected by $τ_{HCO3}$, $[Cl^-]_i$ was only altered at very fast $τ_{HCO3}$. (**b**) Dependency between maximal $[HCO_3^-]_i$ changes and $τ_{HCO3}$ at P_{HCO3} of 0.18 and 0.44 upon a single synaptic stimulation (g_{GABA} = 7.89 nS, τ = 37 ms, initial $[Cl^-]_i$ = 30 mM). Note the sigmoidal dependency between both parameters with halfmaximal responses obtained at $τ_{HCO3}$ of ca. 70 ms. (**c**) Time course of GDP-induced $[Cl^-]_i$ and $[HCO_3^-]_i$ changes (in mM) in the reconstructed CA3 pyramidal neuron (g_{GABA} = 0.789 nS, τ = 37 ms, P_{HCO3} = 0.18, n_{GABA} = 395, initial $[Cl^-]_i$ = 30 mM) at different $τ_{HCO3}$. (**d**) Dependency between GDP-induced $[HCO_3^-]_i$ changes and initial $[Cl^-]_i$ for different $τ_{HCO3}$. The GDP-induced $[HCO_3^-]_i$ changes were virtually absent at $τ_{HCO3}$ of 1 ms (0.15 mM) and amounted to 3.4 mM, 4.5 mM and 4.8 mM for $τ_{HCO3}$ values of 90 ms, 518 ms and 3 s, respectively. (**e**) Dependency between peak GDP-induced depolarizations (g_{GABA} = 0.789 nS, τ = 37 ms, P_{HCO3} = 0.18, n_{GABA} = 395, $τ_{HCO3 = 1s}$) and initial $[Cl^-]_i$ for different pH in the reconstructed CA3 pyramidal neuron. Note that the peak E_m was shifted to depolarized potentials with alkaline pH. (**f–h**) Time course of $[Cl^-]_i$ upon a single synaptic stimulation (g_{GABA} = 1.28 µS, τ = 37 ms, P_{HCO3} = 0.18, initial $[Cl^-]_i$ = 10 mM) determined at the subsynaptic site (red lines) and at the proximal end of an dendrite (blue lines). $[Cl^-]_i$ transients were simulated either in a isolated dendrite (l = 200 µm, shaded lines) or in the identical dendrite connected to a soma (Ø 20 µm, bright lines) at $τ_{Cl}$ of 321 s (**f**), 10 s (**g**) and 1 s (**h**). Note that for $τ_{Cl}$ of 321 s and 10 s the $[Cl^-]_i$ recovers only in the soma-attached configuration, while at $τ_{Cl}$ = 1 s the $[Cl^-]_i$ recovery has a similar decay, suggesting dominant Cl^- elimination via the dendritic membrane. (**i–k**) Distribution of $[Cl^-]_i$ along a 200 µm long dendrite attached to a soma (Ø 20 µm) determined 25 ms, 200 ms, 1 s, and 4 s after the synaptic stimulus (parameters as in a) for $τ_{Cl}$ of 321 s (**i**), 10 s (**j**) and 1 s

(**k**). Note that the $[Cl^-]_i$ in distant parts of the dendrite was substantially lower for $t \geq 1s$ at τ_{Cl} of 10 s and 1 s, as compared to τ_{Cl} of 321 s. Please also note that for $\tau_{Cl} \geq 10s$ $[Cl^-]_i$ was substantial lower in the proximal than in the distal part of the dendrite, suggesting a dominance of diffusional elimination of Cl^- towards the soma under these conditions.
